# Development of a Stress Scale for Siblings of Childhood Cancer Patients

**DOI:** 10.3390/children8040265

**Published:** 2021-03-30

**Authors:** Juyoun Yu, Kyung-Sook Bang, Hyoung Jin Kang

**Affiliations:** 1 Department of Nursing, Changwon National University, Changwon 51140, Korea; hedwigs@changwon.ac.kr; 2 College of Nursing, The Research Institute of Nursing Science, Seoul National University, Seoul 03080, Korea; 3 Department of Pediatrics, Seoul National University College of Medicine, Seoul 03080, Korea; kanghj@snu.ac.kr; 4 Seoul National University Cancer Research Institute, Seoul 03080, Korea; 5 Seoul National University Children’s Hospital, Seoul 03080, Korea; 6 Wide River Institute of Immunology, Hongcheon-gun 25159, Korea

**Keywords:** neoplasm, siblings, psychological stress, psychometrics

## Abstract

Most siblings of childhood cancer patients (SCCP) report not only post-traumatic stress but also daily stresses due to changes in their daily lives. The purpose of this study was to develop a stress scale for SCCP and to examine the validity and reliability of the scale. Based on conceptual analysis, 40 preliminary items were selected. After its content validity was determined by six experts, 37 items were chosen. For the psychometric testing, 125 SCCPs, aged 11–16, were surveyed. Through item analysis and exploratory factor analysis for construct validity, 27 items explained 61.2% of the variance, and they were categorized into six factors. Criterion validity was confirmed by examining the overall correlation with standard instruments according to the age group. Reliability was evaluated using Cronbach’s alpha (0.91) and test-retest correlation (r = 0.597). This self-administered questionnaire with a 4-point Likert-type scale may be useful in clarifying and measuring stress levels in SCCPs.

## 1. Introduction

Childhood cancer is the number one cause of death from disease among Korean children [1]. Childhood cancer is traumatic to both the patient and their family, who are indirectly experiencing the disease, and can cause post-traumatic stress reactions [2,3,4]. Healthy siblings of children with childhood cancer may experience negative emotions, such as anxiety and fear of childhood cancer and its treatment process, jealousy toward their sick sibling who receives all the attention, guilt for feeling jealous, and feelings of isolation as they are excluded from the treatment process [5,6]. These emotions can affect their relationships with peers and their school life [7].

Results of previous studies about siblings of childhood cancer patients (SCCPs) have varied depending on the methodology. While qualitative studies using interviews identified the difficulties of SCCPs, quantitative studies measuring the levels of anxiety, depression, and feelings of isolation in SCCPs using common instruments for children found no significant difference in these levels between normal children and SCCPs [5,7,8]. This may be due to the lack of instruments that can appropriately measure the response of SCCPs to their special situations [4,5]. Furthermore, individually measuring the levels of anxiety, depression, and feelings of isolation in SCCPs only manage to examine certain aspects of their difficulties. Thus, an instrument that can gain an overall understanding of SCCPs’ difficulties is needed.

Existing stress assessment instruments for children measure stress caused by a traumatic event and stress in daily life separately. These instruments cannot measure the overall level of stress in SCCPs as they have experienced both a traumatic event and changes in their daily lives [9]. Thus, this study aimed to develop an instrument that assesses various aspects of stress experienced by SCCPs and verified its validity and reliability.

## 2. Materials and Methods

**Figure 1:**
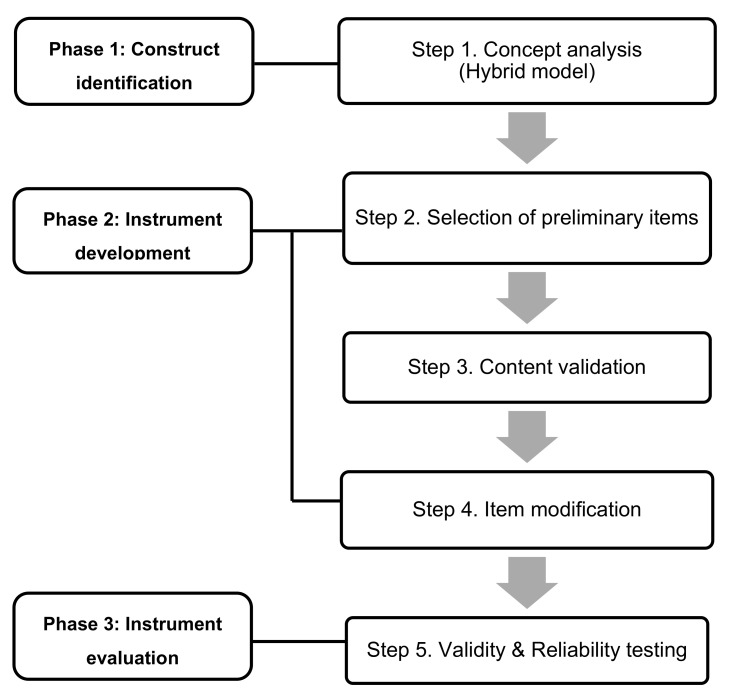
Phases of development of stress scale for siblings of child cancer patients (SCCP).

The present methodological study developed an instrument for measuring stress levels in SCCPs. Following the scale development process proposed by DeVellis [10], an instrument was developed by examining the attributes of the concept, and its validity and reliability were verified (Figure 1). This study was approved by the Institutional Review Board of the research facility (IRB No. 1605/003-005).

### 2.1. Process 1: Instrument Development

A previous study that used the hybrid model to define the concept of stress experienced by SCCPs identified six attributes; fear about childhood cancer, immature coping skills, changes in family relationships, changes in family environment, changes in friendship and school experience, and insufficient social support [11]. Initially, 40 items were created using these attributes as constructs and by referring to existing stress assessment instruments, previous studies, and an interview of 20 SCCPs conducted on previous study of concept analysis. The items were rated on a four-point Likert scale (1 = Not at all, 2 = Somewhat untrue, 3 = Somewhat true, and 4 = Very true) since a scale with an odd number of responses can lead to skewed responses around the median [12].

Two pediatric nursing professors, two pediatric hemato-oncologists, one head nurse from the pediatric hemato-oncology department, and one social worker specializing in rare, incurable pediatric diseases verified the content validity of the initial items. The Scale level- content validity index (S-CVI)/Average score was 0.90, confirming that the items in the instrument were appropriate. Six items with Item-CVI values ≤ 0.78 were removed. Following the experts’ advice, four items were revised, one item was divided into two items to add clarity, and two new items were added. A total of 37 items were selected.

After the instrument’s content validation was completed, five SCCPs were asked about the difficulty of the items and the time required to complete the survey. The SCCPs reported that the items were understandable and that the questionnaire took 3–7 min to complete. The revised instrument consisted of 37 items across the following subdomains: fear about childhood cancer (6 items), immature coping skills (7 items), changes in relationships with family members (10 items), changes in family environment (6 items), changes in friendship and school experience (6 items), and insufficient social support (2 items).

### 2.2. Process 2: Instrument Evaluation

#### 2.2.1. Participants

The inclusion criteria were 11–16-year-old SCCPs who can understand the questions and express their thoughts. SCCPs who had childhood cancer or a chronic disease were excluded. Data were collected from an outpatient hemato-oncology department of a single university hospital which has the biggest children’s hospital in Seoul, Korea for the four months from December 2016 to March 2017. Considering that SCCPs rarely visit the hospital and are young, their participation was accompanied by the presence of their legal guardians. The legal guardians of the SCCPs were informed about the study and discussed with their children whether to participate in the study. Once the SCCPs consented to participate, they were delivered a consent form, questionnaire sheet, information sheet, an envelope to enclose the completed questionnaire to ensure confidentiality, and another envelope for enclosing and sending the provided documents, via their legal guardians. Completed questionnaires were received via mail or directly received from the legal guardians of the SCCPs during a hospital visit. Of the 129 respondents, four who did not meet the age criteria were excluded, and a total of 125 questionnaires were analyzed. Since there is no standard sample size for scale development, the subject-to-variable ratio varies from 3:1 to 20:1 [13]. Although a 10:1 ratio is generally recommended, there was a practical limit to recruiting SCCPs aged 11–16 years.

To examine the test-retest reliability of an instrument, it is necessary to perform a retest using the same instrument at an approximately two-week interval [14]. Twenty participants (16%) consented to the retest. The time until the retest ranged from one–four weeks due to the nature of the data collection (through mail and the participants’ legal guardians).

#### 2.2.2. Data Analysis

Data were analyzed using SPSS Statistics 21. The content validity index (CVI) was calculated during the item development stage for content validation. Construct validity was verified through item assessment and exploratory factor analysis. And criterion validity was measured based on concurrent validity that was established utilizing two existing instruments, which were used with the authors’ permission. The daily hassles scale for school age children (DHSSAC) [15], which was developed to assess daily stress of school age children in Korea, was used as the standard instrument for school age (11–13 years old participants). For the adolescents (14–16 years old participants), the culturally validated version of the perceived stress scale (PSS) by Cohen and colleagues (1983) was used [16].

For item assessment as part of construct validation, the mean, standard deviations, skewness, and kurtosis of each item and the item-score correlation were analyzed. An exploratory factor analysis was performed after confirming that the data were appropriate for a factor analysis via the Kaiser-Meyer-Olkin (KMO) and Bartlett’s test of sphericity. The principal component analysis (PCA) and the varimax method based on orthogonal rotations were used for exploratory factor analysis. Criterion validity was assessed by examining the Pearson’s correlation coefficients between the instrument developed in this study and the DHSSAC or the PSS.

The reliability of the instrument was assessed using Cronbach’s alpha. Test-retest reliability was determined by examining Pearson’s correlations between the initial and second scores.

## 3. Results

### 3.1. Sample Characteristics

Of the 125 participants, 60 participants (48.0%) were boys and 65 (52.0%) were girls. Sixty (48.0%) were attending elementary school, and 65 (52.0%) were attending middle school. The mean age of the participants was 13.89 years. More than half participants (n = 73, 58.4%) were the first child. Twenty-nine (23.2%) and 23 (18.4%) children were the last child and middle child, respectively.

The mean age of the ill siblings was 11.9 years. More than half (n = 72, 57.6%) were the last child, and 35 (28.0%) and 18 (14.4%) were the first child and middle child, respectively. Fifty (40.0%) ill siblings were diagnosed with childhood cancer in the last year, 47 (37.6%) were diagnosed in the last 1–2 years, 21 (16.8%) were diagnosed in the last 2–5 years, and seven (5.6%) were diagnosed over 5 years ago; most ill siblings were diagnosed in the last two years. The mean time after the diagnosis of childhood cancer was 21 months. Most SCCPs perceived that their families had a middle socioeconomic status (n = 104, 83.2%). Only 18 (14.4%) and three (2.4%) perceived that their families had high and low socioeconomic statuses, respectively (Table 1).

### 3.2. Item Analysis

To determine their discriminatory power, each item was assessed for biases based on item analysis (the mean, standard deviations, skewness, kurtosis, item-total correlation, and Cronbach’s alpha if item deleted) (Table 2). The absolute values for skewness and kurtosis did not exceed 2 and 7 for all items, respectively, meaning that normality was satisfied [14].

The correlation between the revised items and scores was analyzed based on the level of contribution of each item [10]. Five items had a correlation coefficient < 0.30 and were deemed to have small contributions to the overall score. After considering the importance of each item, only two items were removed, and a total of 35 items were selected.

### 3.3. Construct Validity

An exploratory factor analysis was conducted on 35 items for construct validation. The KMO value was 0.819 and the p-value from Barlett’s sphericity test was <0.001 (χ^2^ = 2068.961), meaning that the selected items were appropriate for factor analysis. Factors were derived using a PCA model and varimax rotations. The communality of the 35 items was ≥ 0.4; thus, all items were included in the analysis. Factors with eigenvalues ≥ 1.0 were extracted. An additional factor analysis was performed while considering the number of items with factor loadings ≥ 0.40 and the number of items per factor [17]. Eight items were removed through this process; reversed worded items depicting positive situations, such as “I have my own way of soothing myself during difficult times,” and “My family has become closer since my sibling fell ill” were removed, and, finally, a total of 27 items corresponding to six factors were extracted (Table 3). Item 13 had a factor loading ≤0.40 but was included considering its importance. Each item had factor loadings of 0.399–0.827. Of the items extracted from the factor analysis, item 13 (“I feel upset because my family seems to be doing poorly financially due to the medical costs”) and item 22 (“I feel upset because my parents only give my ill sibling special treatment”) were assigned to a more appropriate factor without drastic differences in factor loadings.

The first factor consisted of items related to the changes in home life or school life of SCCPs after the diagnosis of childhood cancer and was named “changes in daily life.” The factor explained 13.9% of the total variance. “Ineffective coping” (Factor 2) consisted of items related to the inability of SCCPs to appropriately cope with the situations related to childhood cancer. The factor explained 12.3% of the total variance. The third factor explained 11.5% of the total variance and was named “worries about ill sibling.” “Changes in relationships with family members,” (Factor 4) consisted of items related to family relationships and reduced activities with family members, accounting for 9.4% of the total variance. The fifth factor, “fear about childhood cancer,” explained 7.7% of the total variance. Lastly, “concealing information” (Factor 6) consisted of items related to the avoidance of SCCPs in talking to others about their sibling’s childhood cancer and accounted for 6.3% of the total variance. The total variance explained by the instrument was 61.2%.

### 3.4. Criterion Validation

For school-age participants, the correlation between the stress scale for SCCP (SSSCCP) developed in this study and the DHSSAC was analyzed (Table 4). There was a significant correlation between the two scales, but the third (worries about ill sibling), fifth (fear about childhood cancer), and sixth (concealing information) factors of SSSCCP have no significant correlations with DHSSA. The correlation coefficient between the two scales was 0.466 (*p* < 0.001).

For adolescents, the correlation between the Korean version of the PSS and the SSSCCP was analyzed (Table 5). The two scales were significantly correlated. Barring the third factor (worries about ill sibling), a significant correlation was found for all factors. The correlation coefficient between the two scales was 0.514 (*p* < 0.001).

### 3.5. Reliability

The scale had a Cronbach’s alpha with a measure of internal consistency of 0.91. Cronbach’s alphas were obtained for each of the six factors: 0.80, 0.82, 0.77, 0.77, 0.66, and 0.70. A significant correlation was found between the first and second measurements with a Pearson’s correlation coefficient of r = 0.597 (*p* = 0.007), confirming the reliability of the scale.

### 3.6. Final Scale Design

A final scale design was confirmed after verifying validity and reliability. The final scale consists of 27 items, each rated on a four-point Likert scale with scores ranging from 1–4 points. Total scores range from 27 to 108 points, with higher scores indicating higher levels of stress.

## 4. Discussion

In this study, a stress scale for SCCPs was developed based on a concept analysis. An inherent attribute of SCCPs’ stress is the “fear of childhood cancer”, which triggers a strong stress response. As a result, siblings experience “changes in daily life”, which have some distinct features that raise difficulties for children. First, SCCPs are children who need to continue growing. Second, they lack the ability to cope effectively. And, lastly, they do not have enough of a support system [11].

“Changes in daily life” (Factor 1) include changes in the surrounding environments, such as home and school, financial changes, and changes in the relationships with relatives, friends, and acquaintances. Childhood cancer brings significant changes to the life of a family. SCCPs in a poorly functioning family experience increased anxiety, loneliness, and feelings of loss and isolation [9,18,19]. A transition into a new situation entails changes in one’s roles and responsibilities [20]. As SCCPs gain more responsibilities within the house, such as helping with household chores and taking care of younger siblings, they have trouble focusing on their academics or maintaining their hobbies [21,22]. It also affects the peer relationships of SCCPs. Though they felt that they receive emotional support from their peers, they do not believe that their peers understand them [21]. Support from others is an important resource that can protect SCCPs as they psychologically adapt to new situations [23,24]. Emotional support enhances SCCPs’ self-esteem, protecting them from the negative impacts of stress [25].

Ineffective coping (Factor 2) results when SCCPs cope with cancer-related situations in an emotional way instead of focusing on the problem, as they are unlikely to have the ability to address the situation [26]. SCCPs do not express their anxiety or concerns as they do not want to burden their busy parents who are going through a difficult time [19]. A previous report indicates that SCCPs show less maladjustment over time and that their awareness of a situation and their coping behaviors can improve [5,18]. However, developing children can experience difficulties because they lack appropriate coping skills or resources. Therefore, providing resources that enhance the coping behaviors of SCCPs and education on effective coping strategies can help reduce their stress.

The third factor is “worries about ill sibling.” Seeing an ill sibling go through changes in their external appearances or behaviors can be a painful experience for SCCPs [19]. This may be because the ill sibling begins to look unfamiliar, and such changes symbolize a deviation from “normal life” [21]. While positive expectations about a sibling’s conditions and treatment can reduce stress [18], worsening of the sibling’s symptoms increases stress, and the siblings of the ill child feel isolated or helpless as they find themselves unable to contribute to the treatment [19].

“Changes in relationships with family members” (Factor 4) may lead to SCCPs feeling isolated and neglected by their parents or ill sibling. The siblings of the ill child want to feel a sense of belongingness as members of the family [19,24] and care more about how much attention they are receiving from the family rather than how poorly the family is functioning [27]. Therefore, parents must be encouraged to give siblings of the ill child adequate amounts of attention.

The fifth factor is “worries about childhood cancer.” SCCPs develop concern as they observe the treatment process of their ill sibling. More importantly, they fear the death of their siblings [21]. SCCPs may show post-traumatic stress reactions due to this strong fear [2,3,4]. Since the lack of accurate information can aggravate fear, it is important to provide SCCPs with accurate information [28,29]. Simultaneously, information about diseases must be carefully provided because some SCCPs who have interpretative (searching for meaning and understanding) and vicarious (relying on the medical specialist) cognitive coping might feel insecure and anxious from the information [18].

“Concealing information” (Factor 6) refers to the reluctance of SCCPs to tell others that their sibling has childhood cancer or talk about it. Adolescent SCCPs intentionally avoid mentioning childhood cancer because their anxiety can be easily triggered by uncertain responses from others [30]. During the interviews, only a few participants could easily talk about their siblings’ childhood cancer. Most participants avoided the topic or did not bring it up themselves as they believed others would not understand them or they were told not to talk about it by their parents. Coping with a situation alone without telling others can be difficult. Thus, it is necessary to provide SCCPs with opportunities to talk about childhood cancer with other SCCPs or healthcare professionals with whom they can mutually empathize.

Through data analysis, items related to SCCPs’ positive reactions to stressful situations were removed. This may be because there is a lower level of understanding among respondents for reverse worded items in self-reported questionnaires [31]. Further research on how stressful situations caused by childhood cancer affect post-traumatic growth of SCCPs is needed.

A limitation of this study is that the sample size was small due to difficulty of participant recruitment. Furthermore, since questionnaires were distributed and retrieved by the SCCPs’ legal guardians due to the difficulty of meeting the SCCPs, the exact time of questionnaire completion, and whether the SCCPs actually completed the questionnaires themselves is unknown. Although an envelope was provided to enclose the questionnaires, it cannot be known whether the questionnaires were completed in a setting where the participants’ responses were kept confidential.

Despite these limitations, this study is meaningful in that it developed an instrument that measures the overall stress levels of SCCPs based on a concept analysis of stress in SCCPs. The instrument developed in this study provides an insight into the stress levels of SCCPs that cannot be revealed by existing stress scales. This scale can be used in survey studies examining a larger sample of SCCPs, or to assess before-and-after effectiveness of intervention programs for SCCPs.

In this study, a scale to measure SCCP’s stress levels was developed and its psychometric properties were tested. This scale allows for a better understanding of SCCPs and may be used to assess the effectiveness of various intervention programs during their development. Further research investigating whether the scale can be used for SCCPs with a wider age range or the siblings of children with other diseases is needed.

## Figures and Tables

**Table 1:** General characteristics of participants and patients.

Variables		Mean ± SD or n (%)
Gender	Boy	60 (48.0)
	Girl	65 (52.0)
Age (year)		13.89 ± 3.00
School	Elementary school	60 (48.0)
	Middle school	65 (52.0)
Birth order	First	73 (58.4)
	Middle	23 (18.4)
	Last	29 (23.2)
Birth order of patient	First	35 (28.0)
	Middle	18 (14.4)
	Last	72 (57.6)
Age of patient (year)		11.90 ± 4.48
Duration since diagnosis (month)		21.06 ± 23.73
	<1 yr	50 (40.0)
	1–2 yrs	47 (37.6)
	2–5 yrs	21 (16.8)
	>5 yrs	7 (5.6)
Perceived socioeconomic status	High	3 (2.4)
	Middle	104 (83.2)
	Low	18 (14.4)

**Table 2:** Item analysis.

	Item Contents	M ± SD	Skewness	Kurtosis	ITC *	Cronbach’s α If Item Deleted
1	I feel upset because my parents do not pay attention to me as much as they used to.	2.22 ± 0.93	0.387	−0.663	0.507	0.903
2	I feel upset about how my ill sibling looks now.	2.59 ± 0.99	−0.287	−0.921	0.497	0.904
3	I feel upset because my family spends less recreational time together now (e.g., family trip, dining, etc.).	2.49 ± 0.98	0.034	−0.988	0.505	0.903
4	I do not want to follow my parents to the hospital because I am scared of hospitals.	1.68 ± 0.78	0.942	0.286	0.451	0.904
5	I feel upset because my family does not talk to each other as much as before.	1.98 ± 0.85	0.508	−0.432	0.552	0.903
6	I feel upset because I can no longer hang out with my ill sibling.	2.58 ± 0.97	−0.052	−0.961	0.303	0.907
7	I am scared of getting cancer myself.	2.01 ± 0.95	0.448	−0.903	0.469	0.904
8	I feel uncomfortable because I cannot eat the food I want because of my ill sibling.	1.97 ± 0.98	0.701	−0.531	0.625	0.901
9	In difficult times, I end up doing nothing because I do not know what to do.	2.04 ± 0.89	0.474	−0.574	0.597	0.902
10	I feel upset because my parents seem to be fighting about the ill sibling.	1.70 ± 0.82	0.865	−0.208	0.586	0.902
11	I am careful around my ill sibling for the fear of accidentally hurting him/her.	2.52 ± 0.95	−0.348	−0.859	0.521	0.903
12	Sometimes, I feel sick for no reason (e.g., headache, stomachache, etc.).	2.13 ± 0.89	0.235	−0.849	0.374	0.906
13	I feel upset because my family seems to be doing poorly financially due to the medical costs.	1.94 ± 0.86	0.590	−0.377	0.576	0.902
14	Talking about a disease scares me.	1.94 ± 0.88	0.682	−0.229	0.559	0.902
15	Sometimes, I feel angry toward my ill sibling.	2.32 ± 0.97	0.066	−1.035	0.406	0.905
16	I feel annoyed because I keep comparing the current situation with the better days in the past.	1.97 ± 0.90	0.608	−0.435	0.721	0.899
17	I do not want to talk about my ill sibling with my teacher.	2.34 ± 0.88	0.127	−0.679	0.371	0.906
18	I feel frustrated because I do not have any friends who can understand and talk about my situation.	1.83 ± 0.82	0.767	0.054	0.436	0.905
19	I feel worried that something bad will happen to my ill sibling.	2.82 ± 0.88	−0.583	−0.203	0.312	0.907
20	I feel upset at myself for not being able to do anything for my ill sibling.	2.26 ± 0.83	0.256	−0.435	0.363	0.906
21	Taking care of household chores often is becoming burdensome.	1.90 ± 0.85	0.841	0.285	0.427	0.905
22	I feel upset because my parents only give my ill sibling special treatment.	1.96 ± 0.99	0.680	−0.667	0.665	0.900
23	I do not want my classmates to find out about my ill sibling.	2.34 ± 0.98	0.234	−0.910	0.383	0.906
24	I feel upset because my living environments have changed due to my sibling’s treatment (e.g., room change, moving, etc.).	1.62 ± 0.84	1.222	0.698	0.475	0.904
25	I feel discontent because I cannot participate in my favorite activities (hobbies, school activities, etc.) as much as I used to because of my ill sibling.	1.79 ± 0.88	0.780	−0.408	0.538	0.903
26	I feel isolated because people close to me (friends, relatives, teachers, neighbors, etc.) seem to only care about my ill sibling.	1.73 ± 0.86	1.107	0.640	0.586	0.902
27	I feel guilty when I get angry at my ill sibling.	2.00 ± 0.93	0.498	−0.734	0.449	0.904

* ITC: item total correlation.

**Table 3:** Factor analysis.

Factor		Items	F1	F2	F3	F4	F5	F6
Factor 1. Changes in daily life	21	Taking care of household chores often is becoming burdensome.	0.768	0.069	0.085	0.130	−0.123	0.024
	26	I feel isolated because people close to me (friends, relatives, teachers, neighbors, etc.) seem to only care about my ill sibling.	0.696	0.249	0.058	0.185	0.166	0.000
	25	I feel discontent because I cannot participate in my favorite activities (hobbies, school activities, etc.) as much as I used to because of my ill sibling.	0.670	0.386	0.027	−0.102	0.140	0.156
	18	I feel frustrated because I do not have any friends who can understand and talk about my situation.	0.667	−0.100	0.016	0.198	0.168	0.183
	24	I feel upset because my living environments have changed due to my sibling’s treatment (e.g., room change, moving, etc.).	0.556	0.064	0.090	0.016	0.400	0.076
	13	I feel upset because my family seems to be doing poorly financially due to the medical costs.	0.409	0.236	0.442	0.116	0.120	0.119
Factor 2. Ineffective coping	15	Sometimes, I feel angry toward my ill sibling.	0.031	0.733	0.109	−0.041	0.260	−0.112
	12	Sometimes, I feel sick for no reason (e.g., headache, stomachache, etc.).	−0.018	0.678	0.095	0.086	0.050	0.069
	8	I feel uncomfortable because I cannot eat the food I want because of my ill sibling.	0.255	0.670	0.085	0.211	0.230	0.116
	10	I feel upset because my parents seem to be fighting about the ill sibling.	0.379	0.597	0.157	0.162	0.022	0.095
	16	I feel annoyed because I keep comparing the current situation with the better days in the past.	0.472	0.523	0.271	0.185	0.218	0.041
	9	In difficult times, I end up doing nothing because I do not know what to do.	0.351	0.399	0.276	0.223	0.173	0.058
Factor 3. Worries about ill sibling	20	I feel upset at myself for not being able to do anything for my ill sibling.	−0.076	0.135	0.784	0.165	−0.118	0.132
	19	I feel worried that something bad will happen to my ill sibling.	−0.030	0.007	0.783	0.010	0.065	0.061
	11	I am careful around my ill sibling for the fear of accidentally hurting him/her.	0.182	0.127	0.635	0.049	0.328	0.070
	2	I feel upset about how my ill sibling looks now.	−0.008	0.076	0.580	0.490	0.176	0.073
	27	I feel guilty when I get angry at my ill sibling.	0.261	0.180	0.534	0.106	0.112	−0.152
Factor 4. Changes in relationships with family members	3	I feel upset because my family spends less recreational time together now (e.g., family trip, dining, etc.).	0.153	0.214	0.157	0.714	−0.030	0.148
	6	I feel upset because I can no longer hang out with my ill sibling.	0.135	−0.293	0.354	0.682	0.101	−0.123
	5	I feel upset because my family does not talk to each other as much as before.	0.056	0.345	0.206	0.637	0.002	0.307
	1	I feel upset because my parents do not pay attention to me as much as they used to.	0.358	0.301	−0.140	0.584	0.240	−0.090
	22	I feel upset because my parents only give my ill sibling special treatment.	0.449	0.487	−0.087	0.470	0.180	0.149
Factor 5. Fear about childhood cancer	4	I do not want to follow my parents to the hospital because I am scared of hospitals.	0.040	0.230	0.028	0.151	0.786	0.131
	14	Talking about a disease scares me.	0.222	0.105	0.421	0.027	0.594	0.228
	7	I am scared of getting cancer myself.	0.235	0.275	0.157	0.034	0.560	−0.016
Factor 6. Concealing information	17	I do not want to talk about my ill sibling with my teacher.	0.003	0.087	0.154	0.108	0.256	0.826
	23	I do not want my classmates to find out about my ill sibling.	0.389	0.039	0.027	0.082	−0.010	0.786
Eigen value			3.741	3.320	3.117	2.544	2.086	1.707
Variance			13.854	12.295	11.545	9.422	7.726	6.322
Cumulative variance			13.854	26.149	37.694	47.115	54.841	61.163

**Table 4:** Correlations between stress scale for siblings of childhood cancer patients (SSSCCP) and daily hassles scale for school age children (DHSSAC) in school-age children (N = 60).

Variables	F1	F2	F3	F4	F5	F6	SSSCCP
DHSSAC	0.580 (<0.001)	0.450 (<0.001)	0.172 (0.189)	0.287 (0.026)	0.177 (0.175)	0.049 (0.710)	0.466 (<0.001)
SSSCCP = Stress Scale for Siblings of Childhood Cancer Patient DHSSAC = Daily Hassles Scale for School Age Children							
F1 = Changes in daily life F2 = Ineffective coping F3 = Worries about ill sibling			F4 = Changes in relationships with family members F5 = Fear about childhood cancer F6 = Concealing information				

**Table 5:** Correlations between SSSCCP and perceived stress scale (PSS) in adolescent (N = 65).

Variables	F1	F2	F3	F4	F5	F6	SSSCCP
PSS	0.571 (<0.001)	0.357 (0.003)	0.242 (0.053)	0.421 (<0.001)	0.406 (0.001)	0.393 (0.001)	0.514 (<0.001)
SSSCCP = Stress Scale for Siblings of Childhood Cancer Patient PSS = Perceived Stress Scale							
F1 = Changes in daily life F2 = Ineffective coping F3 = Worries about ill sibling			F4 = Changes in relationships with family members F5 = Fear about childhood cancer F6 = Concealing information				
